# Immunomodulatory Potential of KS^TM^ in Activating Natural Killer Cells for Cancer Cell Elimination

**DOI:** 10.3390/ph19101525

**Published:** 2026-09-25

**Authors:** Tanyarat Pianmak, Aussara Panya, Natthanich Boonsatit, Lueacha Tabtimmai, Kiattawee Choowongkomon

**Affiliations:** 1 Department of Biochemistry, Faculty of Science, Kasetsart University, Bangkok 10900, Thailand; tanyarat.pia@ku.th; 2 Cell Engineering for Cancer Therapy Research Group, Department of Biology, Faculty of Science, Chiang Mai University, Chiang Mai 50200, Thailand; aussara.pan@cmu.ac.th (A.P.); natthanich1802@gmail.com (N.B.); 3 Department of Biotechnology, Faculty of Applied Science, King Mongkut’s University of Technology North, Bangkok 10800, Thailand

**Keywords:** immunomodulation, NK cell, traditional herbs, non-small cell lung cancer, immunotherapy, TRALL receptor, cancer treatment

## Abstract

**Background/Objectives:** Cancer remains a major cause of mortality worldwide, and resistance to therapy continues to limit treatment success. Improving the susceptibility of cancer cells to immune-mediated killing, particularly by natural killer (NK) cells, may enhance therapeutic outcomes. This study aimed to determine whether natural products can sensitize non-small cell lung cancer (NSCLC) cells to NK cell-mediated cytotoxicity. **Methods:** The cytotoxic effects of natural products on NCI-H1975 cells were assessed using a cell viability assay. NK cell-mediated killing was evaluated in a co-culture system. The expression of MICA/B, Fas, and TRAIL receptors (DR4 and DR5) was analyzed by flow cytometry. **Results:** Treatment with a natural product and NK cells significantly enhanced cancer cell toxicity compared with either treatment alone. Mechanistically, KS markedly increased the expression of TRAIL death receptors on NCI-H1975 cells. Treatment with KS at 62.5 μg/mL increased the proportion of DR4-positive cells from 13.4% to 56.7% and DR5-positive cells from 99.0% to 99.9%. **Conclusions:** These findings indicate that natural products can increase the susceptibility of cancer cells to NK cell-mediated killing by upregulating TRAIL receptor expression and promoting apoptosis. This sensitizing effect may provide a useful strategy to improve the efficacy of cancer immunotherapy.

## 1. Introduction

One of the leading causes of death worldwide and a major challenge to public health systems is cancer. In 2020, more than 10 million deaths were attributed to cancer globally, with approximately 19.3 million new cases reported [1]. Furthermore, the World Health Organization (WHO) has projected that by 2040, the global incidence of cancer will rise to 24.8 million new cases. Cancer cells are abnormal body cells that grow uncontrollably and spread to other parts of the body, ultimately leading to death [2]. In Thailand, lung cancer is the second most common cancer, following liver and bile duct cancers. Non-small cell lung cancer (NSCLC) accounts for approximately 85% of all lung cancer cases, and the current survival rate of patients with NSCLC remains low despite significant advancements in molecular diagnostics and therapeutic strategies [3,4,5]. NSCLC arises from the accumulation of genetic mutations in epithelial cells of the respiratory tract, leading to the loss of growth control, continuous proliferation, evasion of apoptosis, and metastasis to distant organs. Key genetic alterations often involve genes regulating cell growth, including proto-oncogenes and tumor suppressor genes, resulting in aberrant activation of proliferative signaling pathways and tumor development [6]. Major risk factors for NSCLC include exposure to carcinogens particularly cigarette smoke, air pollution, radiation exposure, and genetic susceptibility. These factors induce DNA damage and chronic inflammation in lung tissue, promoting mutation accumulation and tumorigenesis [7]. Current treatment modalities for lung cancer include surgical resection, which is suitable for early-stage localized tumors, radiotherapy, and chemotherapy for advanced-stage disease, as well as targeted therapy and immunotherapy, which have been developed to enhance therapeutic efficacy [8]. However, these treatment strategies still face significant limitations, including drug resistance, recurrence, and severe adverse effects. Therefore, there remains a critical need to develop novel therapeutic approaches that are more specific and safer.

Recently, immunotherapy strategies that stimulate the patient’s immune system have been developed for cancer treatment. Cancer immunotherapy has demonstrated remarkable efficacy in several malignancies, including multiple myeloma and leukemia [9]. Natural killer (NK) cells play a crucial role in tumor immune surveillance. As components of the innate immune system, NK cells serve as a first line of defense by rapidly eliminating cancer cells without prior sensitization. NK cells, first identified in the 1970s [10], are innate lymphocytes with cytotoxic functions that inhibit tumor growth through immune surveillance mechanisms without requiring prior immunological memory. They play a critical role in innate immunity [11]. NK cells recognize target cells through a balance of activating and inhibitory receptors [12]. Under steady-state conditions, NK cell activity is inhibited through recognition of major histocompatibility complex (MHC) class I molecules [13]. A key feature of NK cells is their ability to exert cytotoxicity against tumor cells that lack MHC class I expression, a common immune evasion strategy against CD8+ cytotoxic T cells [14]. This suggests that NK cells and CD8+ T cells function complementarily in tumor immunity [15]. Nevertheless, tumor cells expressing MHC class I can also be eliminated by NK cells through the expression of stress-induced ligands that activate NK cell receptors, including NKG2D, NKp46, and NKp44 [16,17,18]. Among these, NKG2D ligands such as MICA/B are frequently upregulated in human tumor cells under cellular stress conditions, such as DNA damage, thereby activating NK cells for immune surveillance [19]. The ability of NK cells to recognize and attack stressed or pathogen-infected cells provides significant therapeutic potential in cancer, infectious diseases, and autoimmune disorders [20]. Thus, NK cells and CD8^+^ T cells function complementarily in anti-tumor immunity. Importantly, NK cells can also eliminate tumor cells that retain MHC class I expression through engagement of activating receptors that recognize stress-induced ligands. Among these, the NKG2D receptor plays a pivotal role by detecting ligands such as MICA and MICB, which are frequently upregulated in tumor cells under conditions of cellular stress, including DNA damage. Engagement of NKG2D enhances NK cell activation and promotes cytotoxic granule release. In addition to stress ligand recognition, NK cell-mediated killing can proceed through death receptor pathways. Tumor cell expression of Fas receptor (CD95) and TRAIL receptors (DR4 and DR5) renders them susceptible to apoptosis upon interaction with Fas ligand (FasL) or TRAIL expressed on activated NK cells. Upregulation of these surface molecules therefore represents a critical determinant of tumor cell sensitivity to NK cell-induced apoptosis. Collectively, modulation of stress ligands such as MICA/B and death receptors including CD95, DR4, and DR5 may significantly influence tumor immunogenicity and responsiveness to NK cell-mediated cytotoxicity. Despite advances in cancer immunotherapy, limitations such as high treatment costs and adverse effects remain major challenges. Therefore, identifying novel strategies capable of enhancing tumor susceptibility to NK cell-mediated killing may provide complementary approaches to improve current therapeutic outcomes [18,20].

The integration of immunotherapy with natural compounds has emerged as a promising approach to enhance the immune system’s ability to eliminate cancer cells while reducing treatment-related side effects [21]. Bioactive compounds derived from plants are increasingly being developed as alternative cancer therapies. These herbal formulations contain diverse phytochemical components that are considered safe and effective when used alongside conventional cancer treatments. Several Thai herbal formulations have been investigated for their anticancer and immunomodulatory properties, particularly Kerra™, KS™, and Minoza™, which were developed based on traditional Thai medical knowledge and have gained attention in cellular-level research. Kerra™ is an herbal product derived from the ancient Thai medical scripture “Tak-Ka-Si-La” and was traditionally used as an antipyretic remedy. It consists of nine medicinal plants, including *Pterocarpus santalinus*, *Mansonia gagei*, *Schumannianthus dichotomus*, *Momordica cochinchinensis*, *Citrus aurantifolia*, *Combretum quadrangulare*, *Tiliacora triandra*, *Tinospora crispa*, and *Dregea volubilis* [22,23]. Studies have demonstrated that Kerra™ inhibits the proliferation of HCT116 colorectal cancer cells in a dose-dependent manner through regulation of apoptosis-related protein expression [24]. The herbal components within this formulation also exhibit antioxidant, anti-inflammatory, and anticancer activities via multiple mechanisms, including apoptosis induction, inhibition of mutated proteins, and immune modulation. KS™ is another Thai herbal formulation developed according to the four-element theory of traditional Thai medicine. It contains more than 20 medicinal plants, including *Zingiber officinale*, *Derris scandens*, *Mallotus repandus*, and *Piper sarmentosum* [22,23]. Several components in this formulation possess pharmacological properties such as anti-inflammatory, antibacterial, antioxidant, and anticancer activities. Bioactive constituents such as triterpenoids from *Mallotus repandus* and anti-inflammatory compounds from ginger have been reported. Although direct evidence of the combined anticancer effects of the full KS™ formulation remains limited, its herbal constituents may exert synergistic immunomodulatory and antitumor effects. Minoza™ is an herbal formulation aimed at detoxification and immune stimulation. It comprises six medicinal plants, including *Murdannia loriformis*, *Smilax corbularia*, *Phlogacanthus sirindhorniae*, *Parinari anamensis*, *Aloe vera*, and *Glycosmis pentaphylla*. Its major phytochemical constituents include phenols, flavonoids, tannins, alkaloids, and steroids, which exhibit antioxidant, anti-inflammatory, immune-enhancing, and anticancer activities through various mechanisms, including apoptosis induction, free-radical scavenging, and anti-mutagenic effects [25,26,27,28,29,30].

Overall, these three herbal formulations demonstrate biological potential related to anticancer activity through apoptosis induction, antioxidant activity, anti-inflammatory effects, and immune modulation. However, the mechanisms underlying their immunomodulatory effects, particularly on innate immune effector cells such as natural killer (NK) cells, remain insufficiently understood. Moreover, their potential to enhance cancer cell elimination through immune activation lacks detailed molecular evidence.

To the best of our knowledge, this study provides the first evidence that the Thai herbal formulation KS can enhance NK cell-mediated cytotoxicity against NSCLC cells through selective modulation of tumor-cell death receptor expression. NK cells eliminate tumor cells through multiple mechanisms, including death receptor-mediated apoptosis, and strategies that increase tumor susceptibility to these pathways are increasingly being explored to improve NK-based cancer immunotherapy [31,32]. Recent evidence further demonstrates that natural compounds can sensitize cancer cells to TRAIL-induced apoptosis by increasing death receptor expression and activating stress-responsive pathways, including ROS-mediated ER stress and CHOP-dependent DR5 upregulation [33]. Unlike conventional immunomodulatory approaches that primarily enhance NK-cell activation, our findings suggest that KS may act as a tumor-sensitizing agent by increasing DR4 and DR5 expression, thereby potentially enhancing the susceptibility of cancer cells to TRAIL-mediated apoptosis. Importantly, this effect occurred without significant modulation of MICA/B or Fas expression, suggesting a distinct mechanism of action. These findings provide a novel rationale for investigating KS as a potential adjunctive strategy for NK cell-based cancer immunotherapy.

Therefore, the present study aims to investigate the immunomodulatory effects of selected Thai herbal extracts on NK cell activation and to evaluate their potential to enhance NK cell-mediated cytotoxicity against cancer cells. By integrating traditional medicinal knowledge with contemporary immunological approaches, this study seeks to provide mechanistic insight into the development of novel adjunctive cancer therapies.

## 2. Results

### 2.1. Cytotoxicity of Herb Extracts on Lung Cancer

To investigate the effect of the herb extract formulas KERRA, KS, and Minoza on lung cancer cells, cell viability was determined after herb extract treatment. Non-small cell lung cancer NCI-H1975 cells were treated with various concentrations ranging from 62.5 to 1000 µg/mL for 24 and 48 h. The percentage of viable cells was determined relative to that of the untreated control. The result showed that the herb extracts decreased the cell viability of NCI-H1975 cells in a dose-dependent manner (Figure 1A,B). Among them, KS showed the highest toxicity to the cells, followed by KERRA and Minoza with CC50 values of 604.4, 164.5, and 626.7 µg/mL at 48 h after treatment.

### 2.2. Anti-Tumor Activity of NK Cells Against Lung Cancer

The killing assay was performed to evaluate the ability of NK cells to kill lung cancer cells. The cytotoxicity of KHY-G NK cells was determined after 24 h of co-culturing with the cancer cells. The living cancer cells after co-culture were discriminated by detecting the fluorescence signal. The result showed that the viability of cancer cells was reduced to 91.42, 55.31, and 44.12% after co-culture at E:T ratios of 5:1, 10:1, and 20:1, respectively (Figure 2). The data suggests that the cytotoxicity of KHY-G against NCI-H1975 cells is dose-dependent.

### 2.3. The Immunomodulation Effect of Herb Extracts on Enhancing NK Cytotoxicity

To determine the immunomodulation activity of KERRA, KS, and Minoza, NK cells were co-cultured with cancer cells in the presence or absence of the herb extracts. The cancer cell viability was evaluated after 24 of incubation and compared to that of NK treatment alone or extract treatment alone. Among the three extracts, KS improved anti-tumor activity by reducing cancer cell viability compared to NK alone (70.03%) and extract treatment alone (from 85.64%, 76.65%, and 58.96% at concentrations of 125, 250, and 500 µg/mL to 46.02%, 44.17%, and 7.77%, respectively, in the combination treatment). Unfortunately, KERRA and Minoza combined with NK cells caused only slight changes comparing to the individual treatments. This result suggests that KS’s immunomodulation activity improves NK cytotoxicity against lung cancer cells.

### 2.4. Bliss Independence Analysis of NK Cell and Herbal Extract Combination

Bliss independence analysis showed that the combination of NK cells and KS produced synergistic effects at all three tested concentrations. The calculated ΔBliss values were 0.140, 0.095, and 0.335, respectively. The highest synergistic effect was observed at the highest concentration of KS (ΔBliss = 0.335), whereas lower concentrations showed ΔBliss values of 0.140 and 0.095. These results indicate that the combined treatment with NK cells and KS produced greater cytotoxic effects than those expected under Bliss independence (Table 1).

### 2.5. KS Treatment Sensitizes Cancer Cells Through Modulation of Death Receptor Expression

To explore the underlying mechanism contributing to the immunomodulatory activity of KS, we examined alterations in the expression of MICA/B, Fas receptor (FasR), and TRAIL receptors (DR4 and DR5) using flow cytometry. Interestingly, KS treatment significantly increased the expression of TRAIL receptors, whereas no significant changes were observed in MICA/B or FasR expression. Treatment with KS at a concentration of 62.5 μg/mL increased the percentage of DR4- and DR5-positive cells from 13.4% and 99.0 to 56.7% and 99.9%, respectively. Consistent with the increased proportion of receptor-positive cells, the mean fluorescence intensity (MFI) was also markedly elevated following KS treatment. Specifically, the MFI of DR4 increased from 47,495 to 124,218, while DR5 expression increased from 87,165 to 252,242. These findings suggest that KS enhances the expression of TRAIL death receptors, which may increase cancer cell sensitivity to NK cell-mediated cytotoxicity.

## 3. Discussion

Cancer immunotherapy has revolutionized modern oncology by exploiting the immune system to selectively eliminate malignant cells. Among immune effectors, natural killer (NK) cells play a central role in tumor immune surveillance because they can rapidly eliminate transformed cells without prior antigen sensitization [11]. However, many solid tumors, including non-small cell lung cancer (NSCLC), develop resistance to immune-mediated killing through insufficient expression of activating ligands or apoptosis-inducing receptors [34]. Consequently, strategies capable of sensitizing tumor cells to NK cell-mediated cytotoxicity represent an important complementary approach to improve immunotherapy efficacy.

Traditional herbal medicines contain diverse phytochemicals that can modulate immune responses and tumor cell susceptibility to immune attack [35,36]. Nevertheless, their effects on NK–tumor interactions, particularly through regulation of death receptor signaling, remain incompletely understood. Therefore, this study investigated whether Thai herbal formulations (KERRA, KS, and Minoza) can enhance NK cell-mediated killing of lung cancer cells and elucidated the underlying molecular mechanism. This study demonstrated three principal findings. First, all herbal extracts exhibited dose-dependent cytotoxicity toward NCI-H1975 lung cancer cells, indicating intrinsic anti-tumor activity. Among them, KS displayed the strongest cytotoxic potential (Figure 1A,B). Second, NK cells alone exerted effective anti-tumor activity in an effector-to-target ratio-dependent manner, confirming the susceptibility of NCI-H1975 cells to NK-mediated killing (Figure 2). However, the combination treatment revealed an effect exceeding the expected effect under Bliss independence. Bliss analysis further supported this observation, with positive ΔBliss values observed at all three tested KS concentrations (0.140, 0.095, and 0.335), indicating effects greater than those predicted from the individual treatments. The highest ΔBliss value was observed at the highest KS concentration (ΔBliss = 0.335), suggesting that the interaction between KS and NK cells was most pronounced at this concentration. Together, these findings support a synergistic interaction between KS and NK cells rather than a simple additive effect. Third and most importantly, KS significantly enhanced NK cell-mediated cytotoxicity beyond the additive effect of KS and NK cell treatment alone (Figure 3A–C). Mechanistically, this sensitization correlated with a marked upregulation of TRAIL death receptors DR4 and DR5, while MICA/B and Fas expression remained unchanged (Figure 4A–E). These findings indicate that KS promotes tumor elimination primarily through activation of the extrinsic apoptotic pathway rather than through NKG2D-dependent recognition. The direct cytotoxicity observed in all extracts is consistent with numerous phytochemical-based anti-cancer compounds that induce oxidative stress and apoptosis in tumor cells [36]. However, the CC50 values indicate that at sub-cytotoxic concentrations, KS enhanced NK killing without massive direct toxicity. This suggests that the extract acts predominantly as a tumor sensitizer rather than a conventional chemotherapeutic agent. Such sensitization is particularly important because immunotherapy efficacy often depends more on tumor immunogenicity than intrinsic immune potency. NK cytotoxicity depends on a balance between activating ligands and apoptotic susceptibility. Our data showed a classic E:T ratio-dependent killing pattern, supporting intact NK recognition machinery in NCI-H1975 cells.

However, the combination revealed an effect greater than that expected under Bliss independence. Bliss analysis further supported this observation, with positive ΔBliss values observed at all three tested concentrations of KS (0.140, 0.095, and 0.335), indicating effects greater than those predicted from the individual treatments. The highest ΔBliss value was observed at the highest KS concentration (ΔBliss = 0.335), suggesting that the interaction between KS and NK cells was most pronounced at this concentration. Together, these findings support a synergistic interaction between KS and NK cells rather than a simple additive effect. The most striking finding was the selective upregulation of DR4 and DR5 after KS treatment. TRAIL receptors are critical mediators of NK-induced apoptosis, as activated NK cells express TRAIL and induce extrinsic apoptotic signaling upon receptor engagement [37]. Upregulation of DR4/DR5 dramatically enhances tumor susceptibility to immune killing even when activating ligands remain unchanged. This explains why MICA/B levels were not altered while cytotoxicity increased significantly. Mechanistically, death receptor signaling activates caspase-8 and downstream caspase cascade, leading to apoptosis independent of granzyme. Therefore, KS appears to shift NK killing toward receptor-mediated apoptosis rather than perforin-dependent cytolysis. Previous studies on herbal immunomodulators mainly reported macrophage activation or cytokine modulation [36]. Few studies have demonstrated enhancement of NK cytotoxicity through modulation of tumor death receptors. Recent reports showed that natural compounds such as polyphenols and triterpenoids can increase TRAIL sensitivity in cancer cells [38]. Our findings extend this concept by demonstrating that a multi-component herbal formulation can selectively enhance DR4/DR5 expression and improve NK-mediated tumor elimination.

In agreement with our findings, recent studies have highlighted that increasing tumor susceptibility to apoptosis is an effective strategy for improving NK cell-mediated antitumor responses. Rather than directly stimulating NK-cell activity, several natural compounds have been reported to sensitize tumor cells by enhancing death receptor signaling pathways. Recent reviews have emphasized that TRAIL receptor modulation is emerging as an important complementary approach in cancer immunotherapy because it increases tumor responsiveness to immune-mediated apoptosis while minimizing the risk of excessive immune activation [31,32]. These observations support the concept that KS may function primarily as a tumor-sensitizing agent rather than a conventional immune stimulant. The selective upregulation of DR4 and DR5 observed in the present study may be associated with cellular stress-responsive signaling pathways. Previous studies have demonstrated that DR4 and DR5 expression can be induced through p53-dependent mechanisms as well as through reactive oxygen species (ROS)- and endoplasmic reticulum (ER) stress-mediated activation of the PERK–ATF4–CHOP signaling axis, which directly promotes DR5 transcription [31]. In contrast, MICA/B and Fas expression are often regulated through distinct pathways involving DNA damage responses, inflammatory cytokines, and immune surveillance signaling. This difference in regulatory mechanisms may explain why KS selectively enhanced DR4/DR5 expression without significantly affecting MICA/B or Fas receptor levels. Because NCI-H1975 cells harbor TP53 mutations, the relative contribution of p53-dependent signaling remains unclear and warrants further investigation.

Importantly, unlike many compounds that increase NKG2D ligands and risk immune overactivation [39], KS selectively modulated apoptotic susceptibility, potentially reducing systemic immune toxicity. The results suggest that KS functions as an immune sensitizer rather than an immune stimulant. This distinction is clinically relevant because excessive immune activation leads to cytokine release syndrome in immunotherapy [40]. By enhancing tumor vulnerability rather than immune aggressiveness, KS may improve the safety of NK-based therapy. Such tumor-directed sensitization strategies are increasingly recognized as next-generation combination immunotherapy approaches. In lung cancer, resistance to immune checkpoint therapy often involves apoptosis resistance. Upregulating TRAIL receptors could therefore restore immune responsiveness. Several limitations should be acknowledged. First, the study used a single cancer cell line, and tumor heterogeneity may affect the response. Second, NK cell line models differ from primary NK cells in receptor repertoire and activation thresholds. Third, the downstream apoptotic signaling (caspase activation) was not directly confirmed. Fourth, the in vivo pharmacokinetics and immune microenvironment interactions remain unknown.

Therefore, future studies should include primary NK cells, additional tumor models, and animal experiments. In conclusion, this study demonstrates that the Thai herbal formulation KS enhances NK cell-mediated killing of lung cancer cells primarily through upregulation of the TRAIL death receptors DR4 and DR5. Rather than stimulating NK cells directly, the extract increases tumor susceptibility to immune apoptosis. This tumor-sensitizing mechanism represents a promising adjunctive strategy for improving the efficacy and safety of cancer immunotherapy.

## 4. Materials and Methods

### 4.1. Phytochemical Characterization of Kerra Extract

KERRA is a traditional Thai polyherbal formulation composed of nine medicinal plant species, including *Dracaena loureiri* Gagnep., *Tarenna hoaensis* Pit., *Schumannianthus dichotomus* (Roxb.) Gagnep., *Momordica cochinchinensis* (Lour.) Spreng., *Citrus aurantifolia* (Christm.) Swingle., *Combretum quadrangulare* Kurz., *Dregea volubilis* Benth. ex Hook.f., *Tiliacora triandra* Diels., and *Tinospora cordifolia* (Willd.) Hook.f. & Thomson. The phytochemical composition of KERRA has been previously characterized by LC–MS/MS analysis. Briefly, ethanolic extracts were subjected to LC–MS/MS profiling, and compound identification was performed using mzCloud and ChemSpider databases together with Fragment Ion Search (FISh) analysis. The analysis revealed the presence of multiple bioactive phytochemicals in the extract. The KERRA extract used in the present study was prepared using the same formulation and extraction procedure as previously reported. Therefore, the current study focused on the biological evaluation of a chemically characterized extract [2,41,42,43].

### 4.2. Cytotoxicity Assay

The cytotoxicity of the herb extracts KERRA, KS, Minoza was determined using a non-small lung cancer cell line (NCI-H1975). NCI-H1975 was engineered to stably express the red fluorescence protein (RFP) for detection purposes. Briefly, 1 × 10^4^ cells were seeded in 96-well plates a day before the experiment. The herb extracts at concentrations of 0, 62.5, 125, 250, 500, 1000 μg/mL were added to the cells (100 μL per well). The cell viability was determined after 24 and 48 h of incubation by measuring the red fluorescence signal of living cells. The percentage of viable cells was calculated relative to that of the untreated control.

### 4.3. NK-Cancer Cell Co-Culturing and Killing Assay

The cytotoxicity of NK cells against lung cancer cells was evaluated using the killing assay. Briefly, RFP-expressing NCI-H1975 cells were plated in a 96-well format (1 × 104 cells/well). At the time of experiment, the KHY-G NK cells were prepared and co-cultured with cancer cells at effector-to-target (E:T) ratios of 0:1, 5:1, 10:1, and 20:1. After 24 h of incubation, the living NCI-H1975 cells were measured by detecting the RFP signal. The percentage of living cells was calculated relative to the control (E:T ratio of 0:1).

### 4.4. Treatment of Herb Extract to Enhance NK Killing Activity

The ability of the immunomodulation activity of the herb extracts KERRA, KS, and Minoza to improve NK cell cytotoxicity was determined using the killing assay. The RFP-expressing NCI-H1975 cells were plated in a 96-well format (1 × 10^4^ cells/well). At the time of experiment, the KHY-G NK cells were co-cultured with cancer cells at an E:T of 10:1 in the presence or absence of the herb extracts at the indicated concentrations. The cancer cell viability was observed after 24 h of incubation and used to calculate the percentage of living cells.

### 4.5. Bliss Independence Analysis

The interactions between NK cells and each herbal extract (KERRA, KS, and Minoza) were evaluated separately using the Bliss independence model [44]. NK-cell cytotoxicity was assessed at an E:T ratio of 10:1, and each herbal extract was tested at three concentrations. The percentage of target cells killed was calculated as (100 − %) cell viability and converted to fractional values ranging from 0 to 1 before analysis.

For each herbal extract, the expected killing effect of the combination was calculated using the Bliss independence equation:

*Y*_ab,predicted_ = *Y*_a_ + *Y*_b_ − (*Y*_a_ ⋅ *Y*_b_)

where Y_a_ represents the fractional killing effect of NK cells alone and Y_b_ represents the fractional killing effect of the corresponding herbal extract alone at each concentration. The observed fractional killing of the combination *Y*_ab,predicted_ was then compared with the Bliss-predicted value. The Bliss synergy score (ΔBliss) was calculated as:

ΔBliss = Y_observed_ − Y_ab,predicted_

A positive ΔBliss value indicates a synergistic interaction, a value equal to zero indicates an additive interaction, and a negative value indicates an antagonistic interaction [45].

### 4.6. Flow Cytometry

The effect of KS on the surface expression of MICA/B, Fas receptor (CD95) and TRAIL receptor (DR4, DR5) proteins was determined using flow cytometry. Briefly, NCI-H1975 cells (1 × 105 cells/well) were plated in a 12-well format the day before the experiment. The herb extracts at a concentration of 62.5 μg/mL were added to the cells (1 mL per well). The cells were harvested after 24 h of incubation and stained with specific antibodies including APC-conjugated anti-MICA/B antibody (clone 6D4; BioLegend, San Diego, CA, USA), PE-conjugated anti-CD261 (DR4) antibody (clone DJR1; BioLegend, San Diego, CA, USA), APC-conjugated anti-CD262 (DR5) antibody (clone DJ2-4; BioLegend, San Diego, CA, USA), and PE-conjugated anti-CD95 (FasR) antibody (clone DX2; BioLegend, San Diego, CA, USA) for 30 min. The stained cells were washed once with 2% FBS-PBS and were analyzed using a flow cytometer (Beckman Coulter, Inc., Brea, CA, USA), and the data were processed with FlowJo™ v10 Software (FlowJo, Ashland, OR, USA).

### 4.7. Statistical Analysis

Bar graphs show the mean ± standard error of the mean (SEM) from three independent experiments. Statistical analyses were performed using Student’s *t*-test or one-way ANOVA followed by Tukey’s multiple comparisons test, as appropriate, using GraphPad Prism version 8 (GraphPad Software, Inc., La Jolla, CA, USA). A *p*-value > 0.05 was considered non-significant (*ns*). Statistical significance was indicated as follows: * *p* < 0.05, ** *p* < 0.01, *** *p* < 0.001, and **** *p* < 0.0001 compared with the control; # *p* < 0.05, ## *p* < 0.01, ### *p* < 0.001, and #### *p* < 0.0001 compared with the E:T ratio of 10:1.

## 5. Conclusions

The natural products demonstrated the ability to increase the sensitivity of non-small cell lung cancer (NSCLC) NCI-H1975 cells to NK cell-mediated killing. Co-culture experiments showed that cancer cell death was significantly increased compared with treatment with natural products or NK cells alone. Furthermore, mechanistic analysis revealed that the natural product KS significantly increased the expression of the TRAIL receptors DR4 and DR5, which are key mediators of NK cell-induced apoptosis. These findings suggest that the natural product functions as a tumor-sensitizing agent by promoting apoptosis and enhancing immune-mediated elimination of cancer cells. This study highlights a potential strategy for improving cancer immunotherapy. However, the precise mechanisms through which these natural products enhance immune function require further investigation, including their direct effects on NK cells, evaluation in additional cancer models, and further studies to support the development of safe and effective therapeutic approaches.

## Figures and Tables

**Figure 1:**
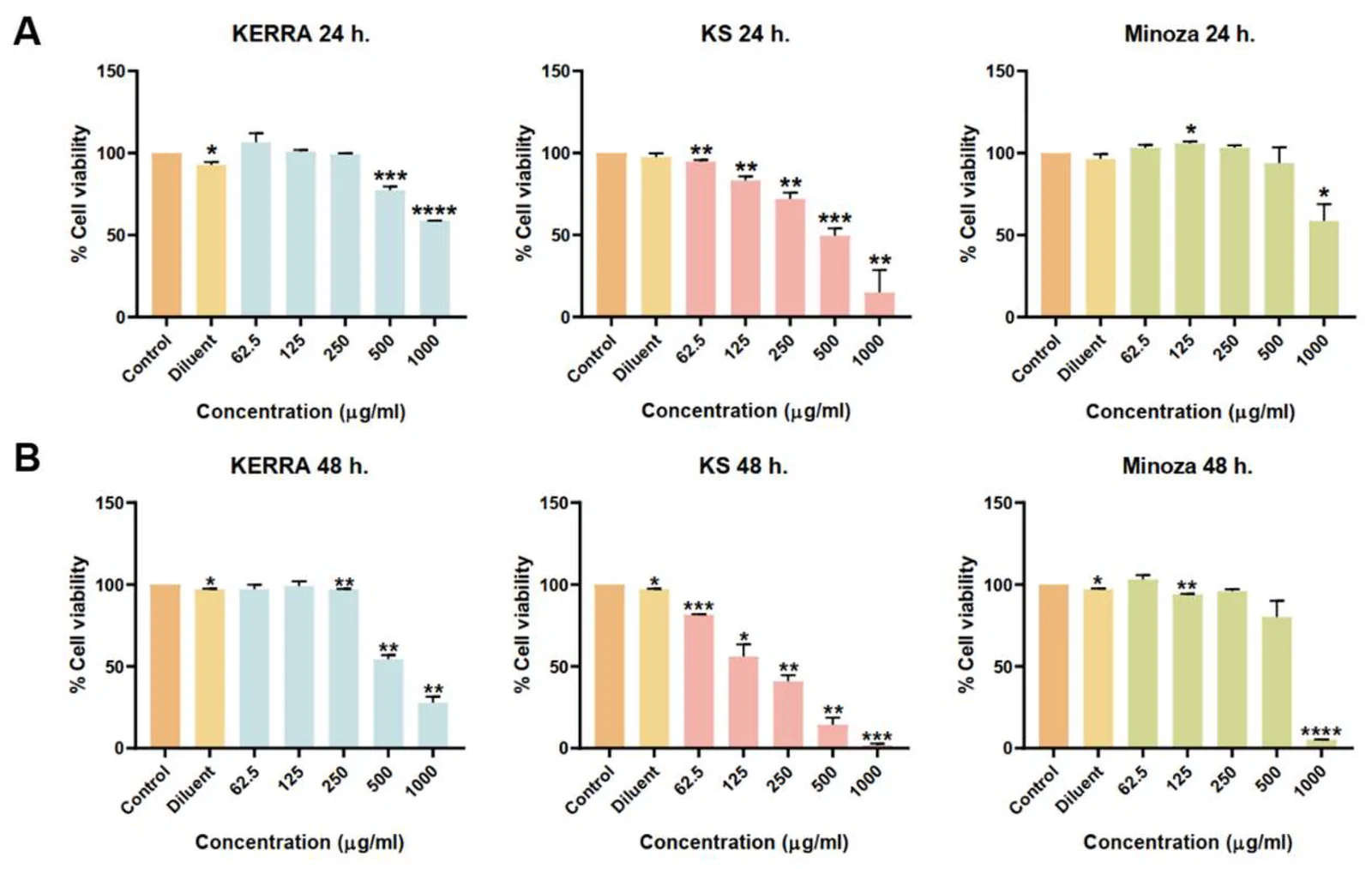
Cytotoxicity of herb extracts. NCI-H1975 cells were treated with various concentrations of KERRA, MS, and Minoza (0–1000 µg/mL) for 24 (**A**) and 48 h (**B**). The percentage of viable cells was measured relative to that of the untreated control (set as 100%). Statistical analysis was performed using one-way ANOVA followed by Tukey’s multiple comparisons test (* *p* < 0.05, ** *p* < 0.01, *** *p* < 0.001, **** *p* < 0.0001 compared with the control; ns, non-significant).

**Figure 2:**
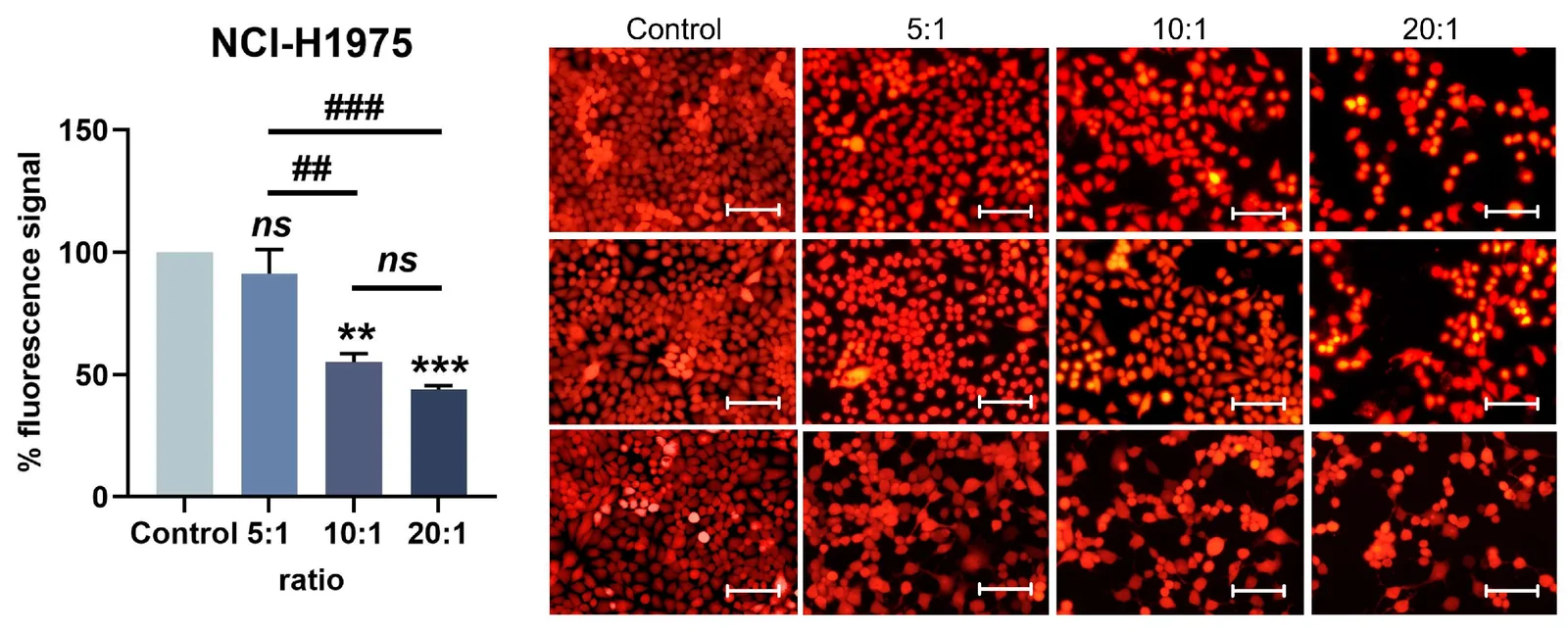
NK cytotoxic activity against lung cancer. The killing assay was performed to evaluate the anti-tumor activity of KHY-G NK cells against NCI-H1975 cells. Cancer cell viability was measured after 24 h of NK cell–cancer cell co-culture at E:T ratios of 0:1, 5:1, 10:1, and 20:1. Statistical analysis was performed using one-way ANOVA followed by Tukey’s multiple comparisons test (** *p* < 0.01, *** *p* < 0.001 compared with the control; ## *p* < 0.01, ### *p* < 0.001 compared with the E:T ratio of 5:1; *ns*, non-significant). Scale bar = 100 µm.

**Figure 3:**
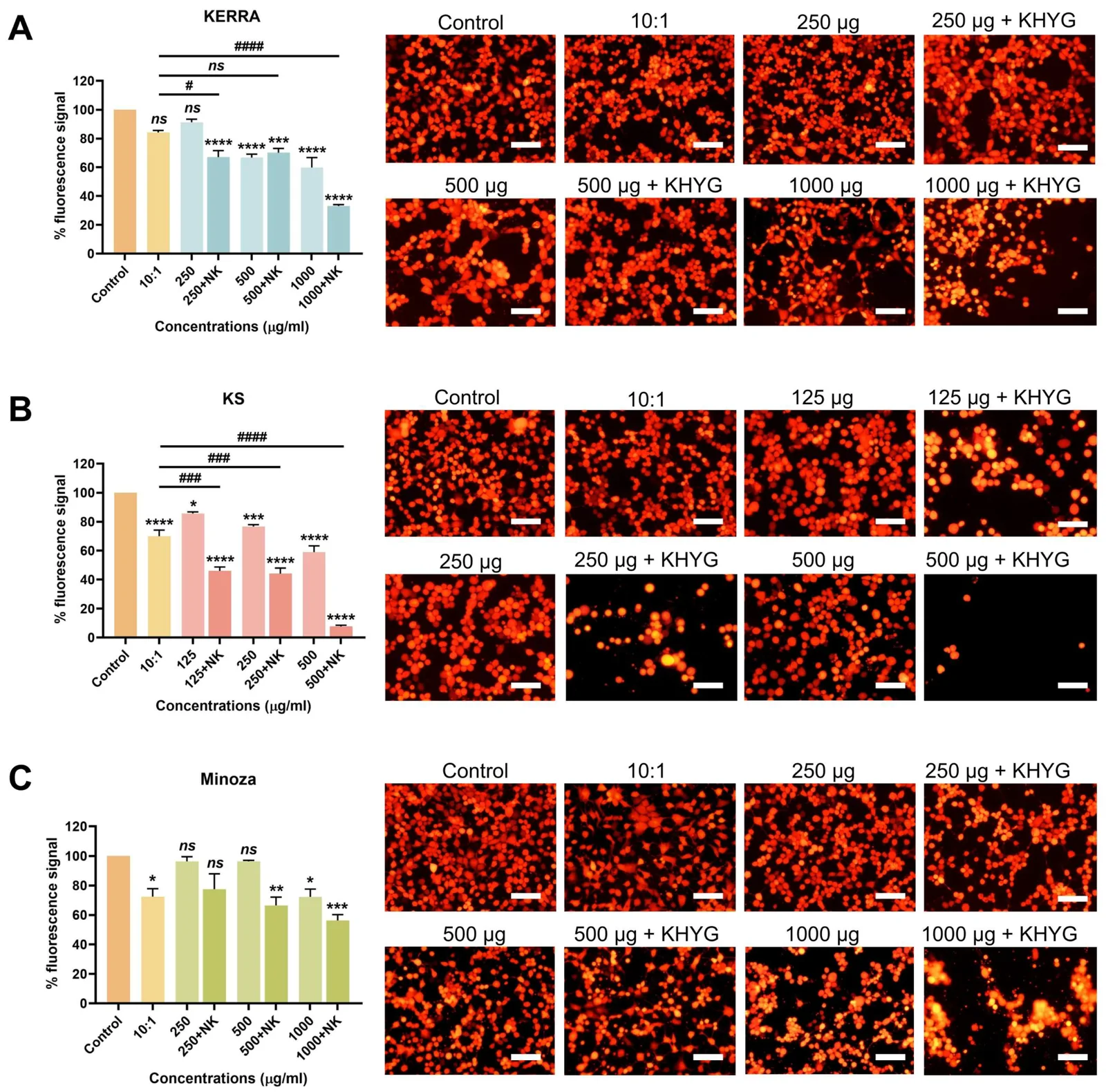
The immunomodulatory activity of herb extracts. The immunomodulatory activity of herb extracts was investigated for their ability to enhance the killing activity of NK cells. Cancer cells and KHY-G cells were co-cultured at different E:T ratios in the presence or absence of KERRA (250, 500, and 1000 µg/mL), KS (125, 250, and 500 µg/mL), and Minoza (250, 500, and 1000 µg/mL). Cancer cell viability was measured after 24 h of co-culture. Statistical analysis was performed using one-way ANOVA followed by Tukey’s multiple comparisons test (* *p* < 0.05, ** *p* < 0.01, *** *p* < 0.001, **** *p* < 0.0001 compared with the control; # *p* < 0.05, ### *p* < 0.001, #### *p* < 0.0001 compared with the E:T ratio of 10:1; ns, non-significant). Scale bar = 100 µm.

**Figure 4:**
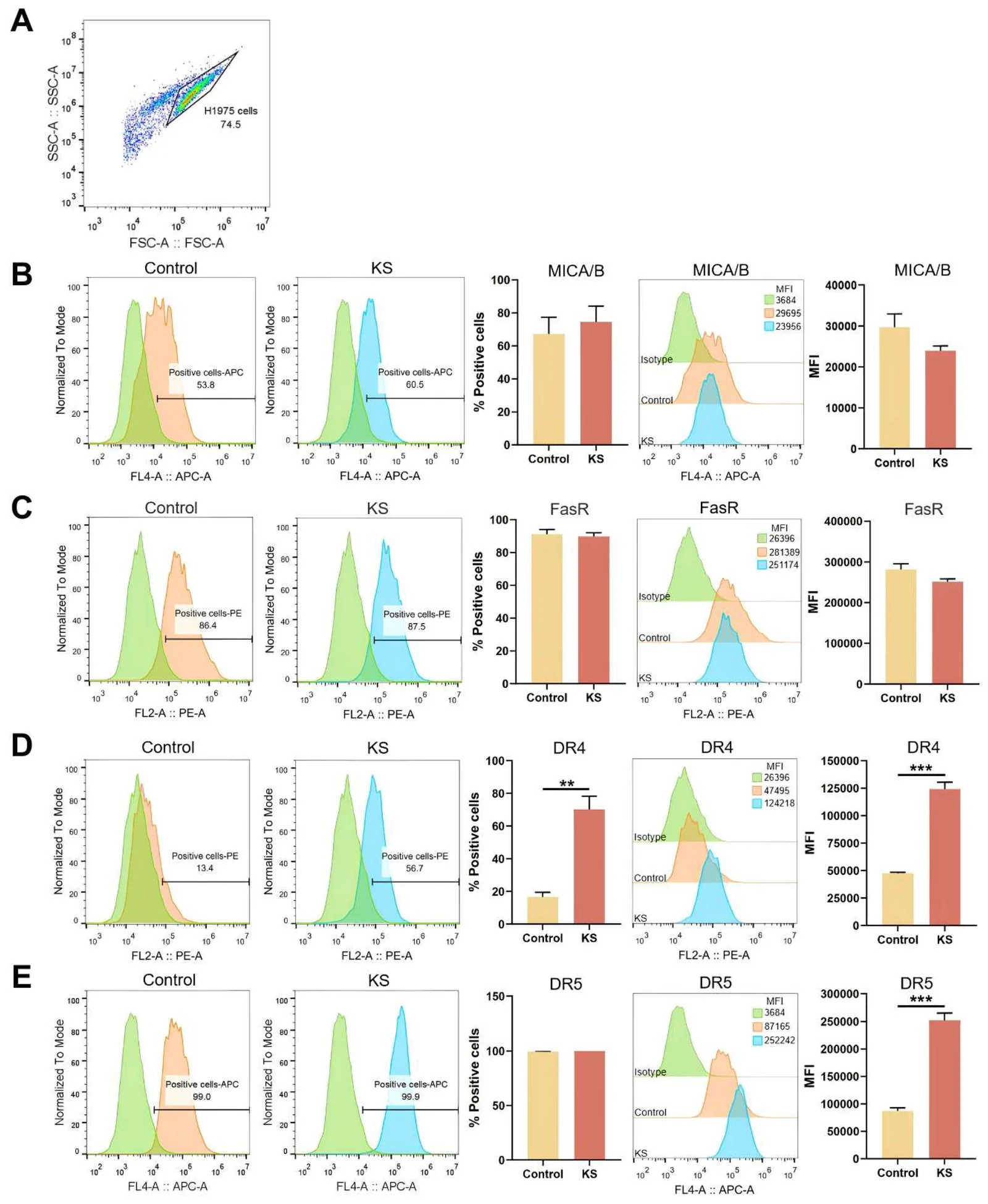
Determination of protein expression after KS extract treatment. Flow cytometry was conducted to assess alterations in NCI-H1975 surface protein expression after 24 h of KS treatment. NCI-H1975 cells were gated according to the gating strategy (**A**). The percentage of positive cells and mean fluorescence intensity (MFI) were analyzed using histograms and bar graphs for MICA/B (**B**), FasR (**C**), DR4 (**D**), and DR5 (**E**), which were compared with the non-treatment control. Statistical analysis was performed using an unpaired *t*-test (** *p* < 0.01, *** *p* < 0.001).

**Table 1:** Bliss synergy scores for the combination of NK cells and an herbal extract.

Herbal Extract	Concentration (µg/mL)	ΔBliss	Interaction
KS	125	0.140	Synergistic
	250	0.095	Synergistic
	500	0.335	Synergistic

## Data Availability

The original contributions presented in this study are included in the article/Supplementary Materials. Further inquiries can be directed to the corresponding author.

## Supplementary Materials

The following supporting information can be downloaded at: https://www.mdpi.com/article/10.3390/ph19101525/s1, Figure S1: Cytotoxicity of herb extracts. A549 cells were treated with the various concentrations of KERRA, MS, and Minoza (0-1,000 μg/mL) for 24 (A) and 48 h (B). The percentage of cell viability was measured relative to that of non-treatment control (set as 100%). (* *p* < 0.05, ** *p* < 0.01, *** *p* < 0.001, **** *p* < 0.0001); Figure S2: NK cytotoxic activity against A549 lung cancer. The killing assay was performed to test the anti-tumor activity of KHY-G NK cell line against A549. The cancer cell viability was observed after NK-cancer cell co-culture for 24 h at the E:T ratios of 0:1, 5:1, 10:1, and 20:1. (* *p* < 0.05, *** *p* < 0.001, **** *p* < 0.000, and scale bar = 100 micrometer); Figure S3: The immunomodulation activity of herb extracts in A549 cells. A549 and KHY-G cells were co-cultured at the E:T ratios of 10:1 in the presence of absence of KERRA (250, 500 µg/mL), KS (125, 250 µg/mL), and Minoza (500, 1000 µg/mL). The cancer cell viability was observed after 24-h co-culture.; Figure S4: Cytotoxicity of herb extracts in NCI-H1975 cells. NCI-H1975 cells were treated with serial concentrations of KERRA (A), KS (B), and Minoza (C) for 48 h. Cell viability was determined using the Crystal Violet staining assay. Dose–response curves were generated by nonlinear regression to determine the CC50 values.; Figure S5: Original microscopy images corresponding to Figure 3. Original full-field microscopy images showing the immunomodulation activity of herb extracts on the killing activity of NK cells. Cancer cells and KHY-G cells were co-cultured at an effector-to-target (E) ratio of 10:1 in the presence or absence of the indicated concentrations of herb extracts. (A) KERRA at 250, 500, and 1000 µg/mL; (B) KS at 125, 250, and 500 µg/mL; and (C) Minoza at 250, 500, and 1000 µg/mL. Images were acquired under the same experimental conditions as those presented in Figure 3. Scale bars, 100 µm.; Figure S6: Thai Food and Drug Administration (FDA) certification document for the KS herbal formulation.; Figure S7: Chromatogram of extracted metabolites profiling. (A) Independent experiment 1 and (B) Independent experiment 2. Chromatograms from two independent experiments are presented in Figures S7A and S7B. The chromatograms of independent experiments 1 and 2 demonstrated consistency and reproducibility. The reproducibility of the highest peak at m/z = 177.0916, eluting at approximately 14.2 min, was observed across two independent LC-MS runs. Moreover, this peak exhibited excellent symmetry and consistency across the two experimental batches, with a compound coefficient of variation per sample batch of 5% Table S1: Composition and ingredients of the KS herbal formulation. The table presents the individual herbal ingredients.

## Abbreviations

The following abbreviations are used in this manuscript:

NSCLC	Non-small cell lung cancer
NK cells	Natural killer cells
TRAIL	Tumor necrosis factor-related apoptosis-inducing ligand
DR4	Death receptor 4
DR5	Death receptor 5
MICA/B	MHC class I chain-related proteins A/B
FasR	Fas receptor (CD95)
