# Supplementary material for: Immunomodulatory Potential of KSTM in Activating Natural Killer Cells for Cancer Cell Elimination
Source: Pharmaceuticals (Basel). 2026 Sep 25;19(10):1525. doi: 10.3390/ph19101525 (PMC13708112; doi:10.3390/ph19101525)
Supplement: Supplementary file 1 [file pharmaceuticals-19-01525-s001.zip › pharmaceuticals-4469554-supplementary.pdf]

**Immunomodulatory Potential of KSTM in Activating Natural Killer Cells for Cancer Cell Elimination**

**Supplementary data**

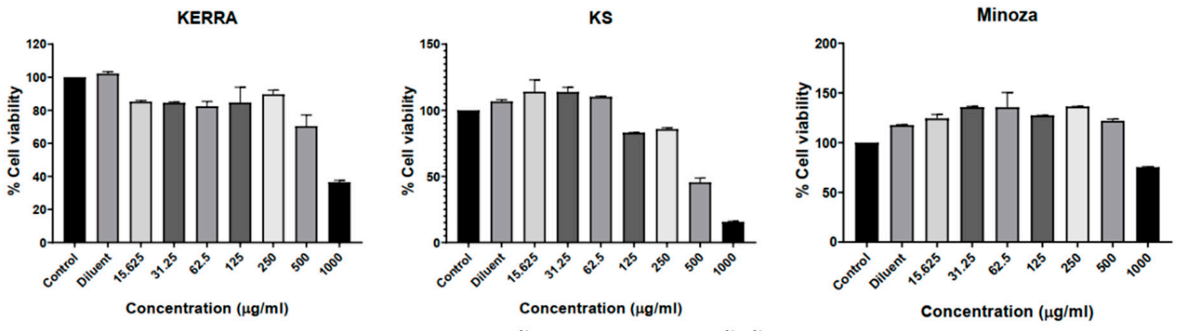


**Figure S1 Cytotoxicity of herb extracts.** A549 cells were treated with the various concentrations of KERRA, MS, and Minoza (0-1,000 µg/mL) for 24 (A) and 48 hours (B). The percentage of cell viability was measured relative to that of non-treatment control (set as 100%). (*p < 0.05, **p < 0.01, ***p < 0.001, ****p < 0.0001)

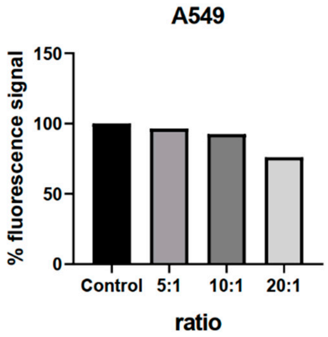


**Figure S2 NK cytotoxic activity against A549 lung cancer.** The killing assay was performed to test the anti-tumor activity of KHY-G NK cell line against A549. The cancer cell viability was observed after NK-cancer cell co-culture for 24 hours at the E:T ratios of 0:1, 5:1, 10:1, and 20:1. (*p < 0.05, ***p < 0.001, ****p < 0.000, and scale bar = 100 micrometer)

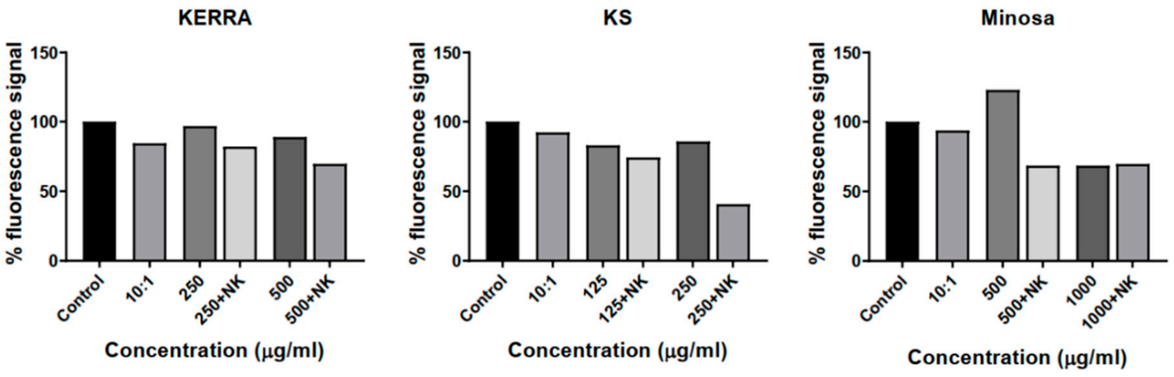


**Figure S3 The immunomodulation activity of herb extracts in A549 cells.** A549 and KHY-G cells were co-cultured at the E:T ratios of 10:1 in the presence of absence of KERRA (250, 500μg/mL), KS (125, 250μg/mL), and Minoza (500, 1000 μg/mL). The cancer cell viability was observed after 24-hour co-culture.

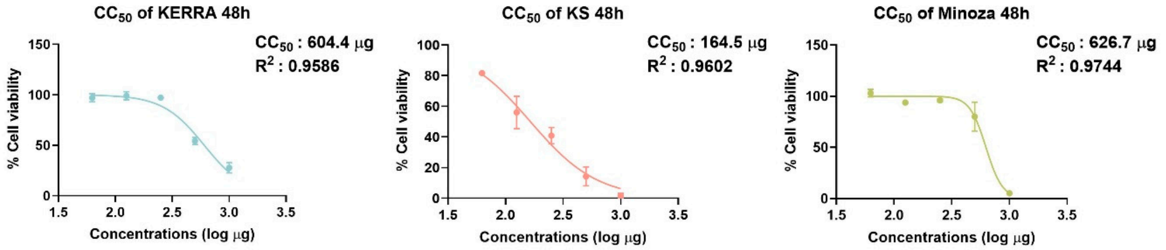


**Figure S4. Cytotoxicity of herb extracts in NCI-H1975 cells.** NCI-H1975 cells were treated with serial concentrations of KERRA (A), KS (B), and Minoza (C) for 48 h. Cell viability was determined using the Crystal Violet staining assay. Dose–response curves were generated by nonlinear regression to determine the $CC_{50}$ values.

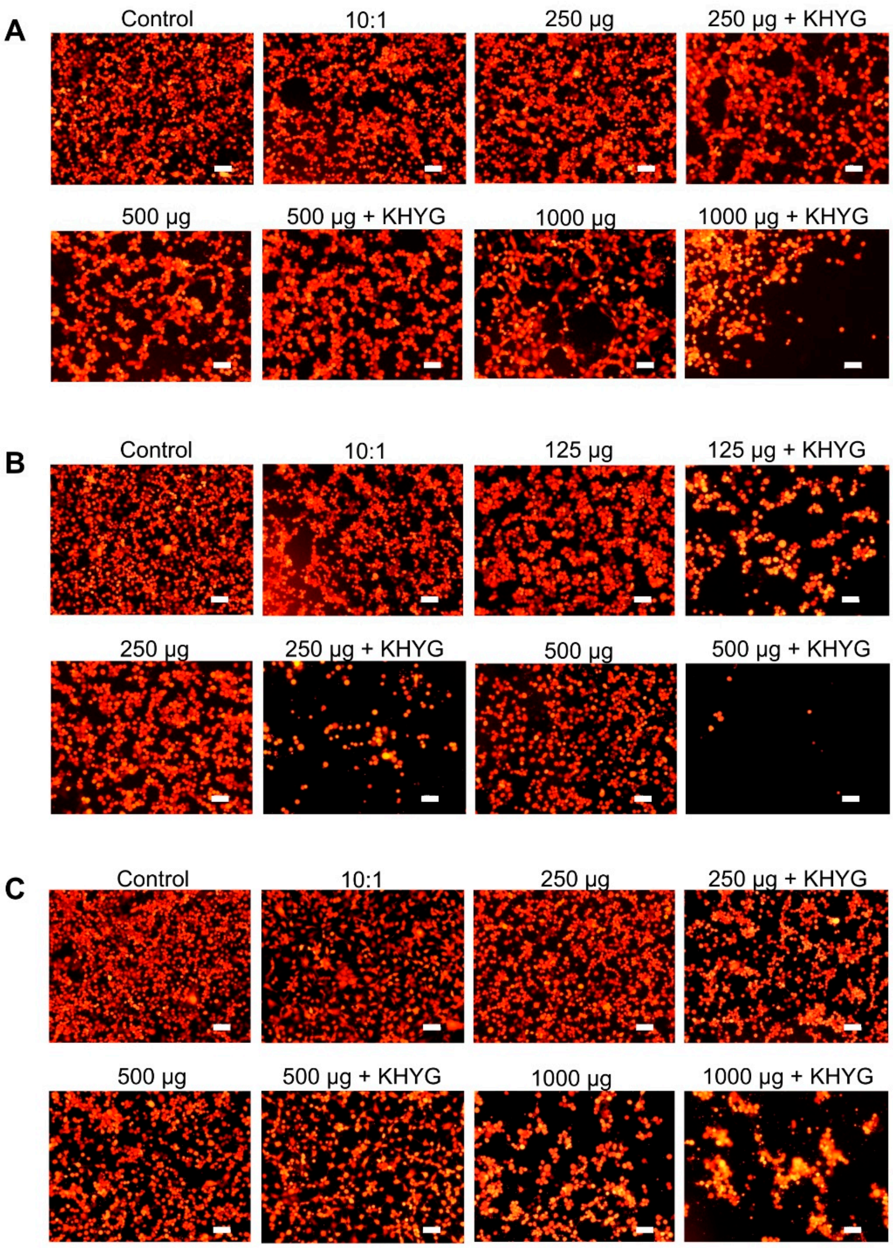


**Figure S5 Original microscopy images corresponding to Figure 3.** Original full-field microscopy images showing the immunomodulation activity of herb extracts on the killing activity of NK cells. Cancer cells and KHY-G cells were co-cultured at an effector-to-target (E) ratio of 10:1 in the presence or absence of the indicated concentrations of herb extracts. (A) KERRA at 250, 500, and 1000 µg/mL; (B) KS at 125, 250, and 500 µg/mL; and (C) Minoza at 250, 500, and 1000 µg/mL. Images were acquired under the same experimental conditions as those presented in Figure 3. Scale bars, 100 µm.

**Ingredients of KS™**

| No. | Ingredients | Scientific Name |
|---|---|---|
| 1 | PIPER RETROFRACTUM VAHL. | PIPER RETROFRACTUM VAHL. |
| 2 | ZINGIBER OFFICINALE ROSCOE. | ZINGIBER OFFICINALE ROSCOE. |
| 3 | PIPER SARMEMTOSUM ROXB. | PIPER SARMEMTOSUM ROXB. |
| 4 | PIPER RIBESIOIDES WALL. | PIPER RIBESIOIDES WALL. |
| 5 | PLUMBAGO INDICA L. | PLUMBAGO INDICA L. |
| 6 | MALLOTUS REPANDUS (ROTTLET) MULL. ARG. | MALLOTUS REPANDUS (ROTTLET) MULL. ARG. |
| 7 | ANAXAGOREA LUZONENSIS A. GRAY. | ANAXAGOREA LUZONENSIS A. GRAY. |
| 8 | SOLORI SCANDENS (ROXB.) SIRICH & ADOMA. | SOLORI SCANDENS (ROXB.) SIRICH & ADOMA. |
| 9 | PIPER NIGRUM L. | PIPER NIGRUM L. |
| 10 | FICUS VILLOSA BLUME. | FICUS VILLOSA BLUME. |
| 11 | BOESENBERGIA ROTUNDA (L.) MANSF. | BOESENBERGIA ROTUNDA (L.) MANSF. |
| 12 | SENNA GARRETTIANA (CRAIB) IRWIN & BARNEBY. | SENNA GARRETTIANA (CRAIB) IRWIN & BARNEBY. |
| 13 | ZINGIBER MONTANUM (J.KOENIG) LINK EX A. DIETR. | ZINGIBER MONTANUM (J.KOENIG) LINK EX A. DIETR. |
| 14 | CAMPHOR | CAMPHOR |
| 15 | MYRISTICA FRAGRANS HOUTT. | MYRISTICA FRAGRANS HOUTT. |
| 16 | SYZYGIUM AROMATICUM (L.) MERR. & L. M. PERRY. | SYZYGIUM AROMATICUM (L.) MERR. & L. M. PERRY. |
| 17 | ARORUS CALAMUS L. | ARORUS CALAMUS L. |
| 18 | ZINGIBER ZERUMBET (L.) ROSCOE EX SM | ZINGIBER ZERUMBET (L.) ROSCOE EX SM |
| 19 | AMOMUM KRERVANH PIERRE EX GAGNEP. | AMOMUM KRERVANH PIERRE EX GAGNEP. |
| 20 | PIPER NIGRUM L. | PIPER NIGRUM L. |
| 21 | GLYCYRRHIZA GLABRA L. | GLYCYRRHIZA GLABRA L. |
| 22 | CINNAMOMUN BEJOLGHOTA (BUCH.-HAM.) SWEET. | CINNAMOMUN BEJOLGHOTA (BUCH.-HAM.) SWEET. |
| 23 | MYRISTICA FRAGRANS HOUTT. | MYRISTICA FRAGRANS HOUTT. |
| 24 | RHEUM PALMATUM L. | RHEUM PALMATUM L. |
| 25 | JASMINUM SAMBAC (L.) AITON. | JASMINUM SAMBAC (L.) AITON. |
| 26 | MIMUSOPS ELENGI L. | MIMUSOPS ELENGI L. |
| 27 | MESUA FERREA L. | MESUA FERREA L. |
| 28 | MAMMEA SIAMENSIS (MIQ.) T. ANDERSON. | MAMMEA SIAMENSIS (MIQ.) T. ANDERSON. |
| 29 | NELUMBO NUCIFERA GAERTN. | NELUMBO NUCIFERA GAERTN. |

Table S1 : Composition and ingredients of the KS herbal formulation. The table presents the individual herbal ingredients

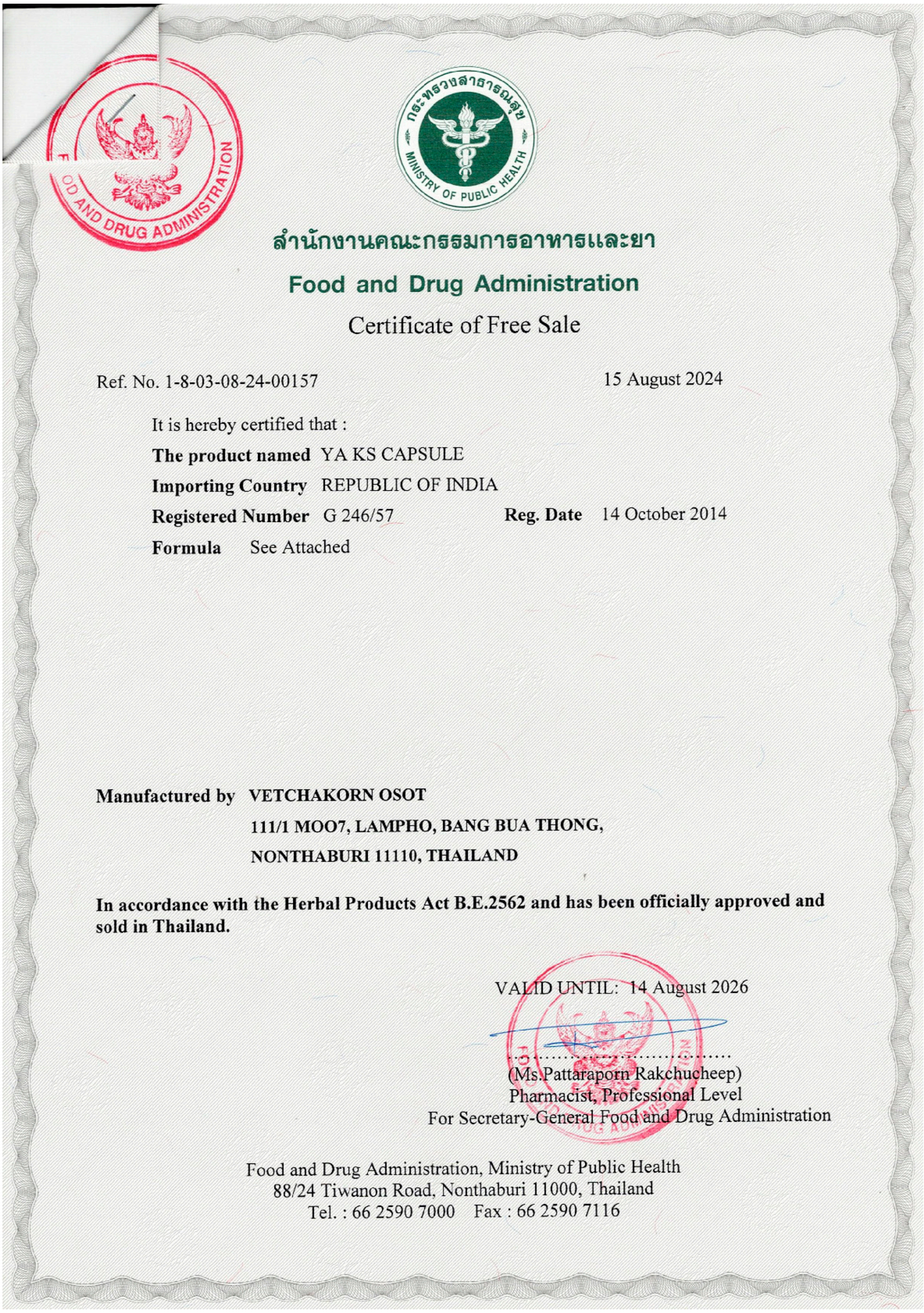

สำนักงานคณะกรรมการอาหารและยา

**Food and Drug Administration**

Certificate of Free Sale

Ref. No. 1-8-03-08-24-00157 15 August 2024

It is hereby certified that :

**The product named** YA KS CAPSULE

**Importing Country** REPUBLIC OF INDIA

**Registered Number** G 246/57 **Reg. Date** 14 October 2014

**Formula** See Attached

**Manufactured by** **VETCHAKORN OSOT**
**111/1 MOO7, LAMPHO, BANG BUA THONG,**
**NONTHABURI 11110, THAILAND**

**In accordance with the Herbal Products Act B.E.2562 and has been officially approved and sold in Thailand.**

VALID UNTIL: 14 August 2026

(Ms.Pattaraporn Rakchucheep)
Pharmacist, Professional Level
For Secretary-General Food and Drug Administration

Food and Drug Administration, Ministry of Public Health
88/24 Tiwanon Road, Nonthaburi 11000, Thailand
Tel. : 66 2590 7000 Fax : 66 2590 7116

Figure S6: Thai Food and Drug Administration (FDA) certification document for the KS herbal formulation.

(A) (B)

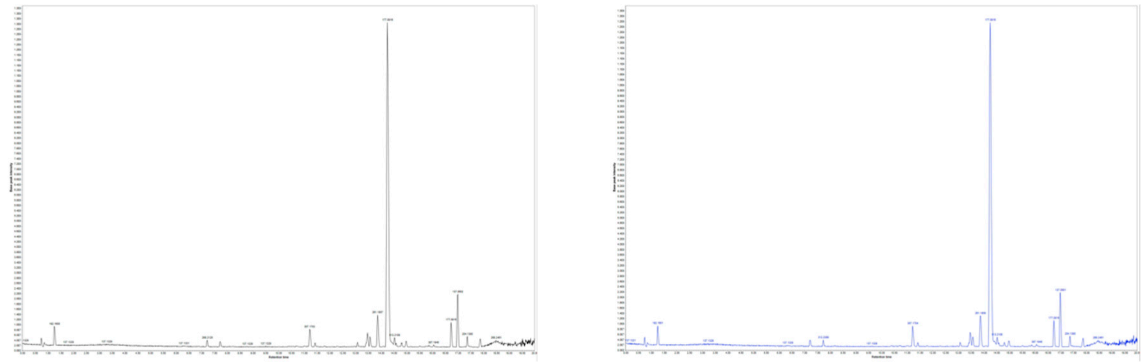

**Figure S7:** Chromatogram of extracted metabolites profiling. (A) Independent experiment 1 and (B) Independent experiment 2. Chromatograms from two independent experiments are presented in Figures S5A and S5B. The chromatograms of independent experiments 1 and 2 demonstrated consistency and reproducibility. The reproducibility of the highest peak at m/z = 177.0916, eluting at approximately 14.2 minutes, was observed across two independent LC-MS runs. Moreover, this peak exhibited excellent symmetry and consistency across the two experimental batches, with a compound coefficient of variation per sample batch of 5%
